# Reference Intervals and Prediction Equation for Sustained Maximal Inspiratory Pressure in Healthy Adults

**DOI:** 10.7759/cureus.112692

**Published:** 2026-07-15

**Authors:** Takuya Ujikawa, Hiroki Sato, Sho Takahashi, Hiromichi Metani, Kozo Hanayama

**Affiliations:** 1 Department of Physical Therapy, Kawasaki University of Medical Welfare, Kurashiki, JPN; 2 Department of Rehabilitation Center, Kawasaki Medical School Hospital, Kurashiki, JPN; 3 Department of Rehabilitation Medicine, Kawasaki Medical School, Kurashiki, JPN

**Keywords:** healthy adults, inspiratory muscle performance, prediction equation, reference interval, sustained maximal inspiratory pressure

## Abstract

Introduction

Sustained maximal inspiratory pressure (SMIP), obtained using the test of incremental respiratory endurance, has been proposed as an index of inspiratory muscle performance reflecting both force generation and the ability to sustain inspiratory effort. However, reference intervals and prediction equations for SMIP in healthy adults have not been sufficiently established. This study aimed to establish sex- and age-specific reference intervals for SMIP, identify factors associated with SMIP, and develop a practical prediction equation.

Methods

This cross-sectional study included healthy adults aged 18 years or older. After exclusion of participants with respiratory or neuromuscular disease and those with missing data, 439 participants were included and stratified by sex and age (<35 and ≥35 years). Reference intervals were calculated for each partition using a nonparametric percentile method. Univariate linear regression analyses were performed to examine factors associated with SMIP, followed by multiple linear regression analyses to develop a prediction equation using age, sex, height, and weight.

Results

SMIP was highest in younger males and lowest in older females. The 95% reference intervals were 383.2-1209.9 pressure time units (PTU) in males aged <35 years, 141.0-1222.0 PTU in males aged ≥35 years, 109.5-667.8 PTU in females aged <35 years, and 97.3-617.1 PTU in females aged ≥35 years. In multivariable analysis, age, sex, and height were independent predictors of SMIP, whereas weight was not independently associated with SMIP after adjustment. The final model explained 56.9% of the variance in SMIP (adjusted R^2^ = 0.565; SEE = 171.20 PTU).

Conclusions

This study established sex- and age-specific reference intervals and a simple and practical prediction equation for SMIP in healthy adults. These findings provide a basis for the quantitative interpretation of SMIP and may support its broader clinical application.

## Introduction

Assessment of respiratory muscle function is important not only in respiratory diseases but also in the evaluation of cardiovascular diseases, neuromuscular disorders, and age-related decline in physical function, because respiratory muscle dysfunction may contribute to ventilatory limitation, dyspnea, and reduced exercise capacity [1-4]. Among the available measures, maximal inspiratory pressure (MIP) is widely used as a representative index of inspiratory muscle strength and reflects the maximal pressure generated during inspiration. It has been applied in respiratory, cardiovascular, and neuromuscular disorders, as well as in age-related decline in physical function, and standardized measurement procedures have been described in international statements [1,2]. Interpretation of MIP also requires reference values and prediction equations that account for the effects of age, sex, and body size, and studies establishing such reference values have been conducted in various countries and populations [5-7].

However, MIP reflects the maximal pressure that the inspiratory muscles can generate instantaneously and does not fully capture the extent to which inspiratory effort can be sustained over time [1]. To address this limitation, sustained maximal inspiratory pressure (SMIP), obtained using the Test of Incremental Respiratory Endurance (TIRE), has been proposed as an index that integrates inspiratory pressure over the entire inspiratory duration and thus more comprehensively reflects both inspiratory muscle force generation and the ability to sustain that effort [8,9].

Recent studies in patients with chronic obstructive pulmonary disease have shown that SMIP has high reproducibility and validity and may reflect functional status more readily than MIP [8-10]. Formiga et al. reported that TIRE-derived measures of MIP, SMIP, and inspiratory duration showed good test-retest reliability, and that SMIP was associated with the six-minute walk distance and dyspnea, whereas such associations were limited for MIP [9]. Nevertheless, several issues must be addressed before SMIP can be more widely applied in clinical practice. First, reference intervals for healthy adults have not been sufficiently established, making it difficult to determine whether an individual's measured value falls within the expected range. Second, factors associated with SMIP, particularly demographic and anthropometric variables, such as age, sex, height, and weight, have not been fully clarified. Because reference values and prediction equations for MIP vary across populations [5-7,11], a similar population-specific evaluation is also needed for SMIP. Establishing reference intervals in healthy adults is an essential first step before SMIP can be interpreted in clinical populations or used to identify abnormal inspiratory muscle performance.

In addition to reference intervals, the clinical interpretation of SMIP also requires prediction equations tailored to individual characteristics. For MIP, several prediction equations incorporating age, sex, height, and weight have been proposed [5,6,11], and comparison of measured values with predicted values or the lower limit of normal is useful for identifying reduced respiratory muscle function and interpreting individual measurements in clinical settings [5,11-13]. Likewise, for SMIP, the establishment of prediction equations based on readily available demographic and anthropometric variables may facilitate interpretation of measured values and enhance its clinical utility in the assessment of respiratory muscle function and the evaluation of intervention effects.

Accordingly, the purpose of this study was to establish reference intervals for SMIP in healthy adults, to examine factors associated with SMIP, and to develop a prediction equation using readily available variables. By doing so, we aimed to provide a basis for the quantitative interpretation of SMIP and to promote the clinical application of this index in the assessment of respiratory muscle function.

## Materials and methods

Study design and participants

This cross-sectional study included healthy adults stratified by sex and age. Participants were classified into four groups: males aged <35 years, males aged ≥35 years, females aged <35 years, and females aged ≥35 years. Partitioning by sex was prespecified because respiratory muscle performance is influenced by sex-related differences in body size and muscle characteristics. Partitioning by age was also considered appropriate because inspiratory muscle performance may change with aging. The age cut-off of 35 years was selected with reference to the policy framework of the Ministry of Health, Labour and Welfare in Japan, in which this age is considered a key milestone for comprehensive adult health assessment within the statutory health screening framework, and was therefore adopted as a practical categorization for age-stratified estimation of reference intervals rather than as an established physiological threshold for SMIP. Written informed consent was obtained from all participants. All procedures were approved by the ethics committees of Kawasaki Medical School and Kawasaki Medical School Hospital (approval no. 5573-02).

Measurements

Participant characteristics included age, height, weight, body mass index (BMI), skeletal muscle mass index (SMI), percent vital capacity (%VC), forced expiratory volume in one second as a percentage of forced vital capacity (FEV_1_/FVC), and maximal inspiratory pressure (MIP). SMIP was measured using TIRE with a PrO2Fit device (PrO2 Health Incorporated, Smithfield, RI, USA) in the sitting position. The device provides a real-time pressure-time graph for immediate monitoring of inspiratory pressure [14]. Participants were instructed to perform a maximal and sustained inspiratory effort through the mouthpiece following full expiration, with visual feedback provided by the real-time waveform. The maneuver automatically ended when inspiratory pressure returned to baseline. Measurements were performed twice at one-minute intervals, and the highest SMIP value was used for analysis. If the difference in MIP between the first two measurements exceeded 20%, a third measurement was performed [15]. SMIP was used as the primary outcome and was expressed in pressure-time units (PTU).

Statistical analysis

Statistical analysis was performed using SPSS Statistics version 31 (IBM Corp., Armonk, NY, USA). Reference intervals for SMIP were calculated separately for the four sex- and age-defined partitions using a nonparametric percentile method consistent with CLSI C28-A3c recommendations [16]. The lower and upper reference limits were defined as the 2.5th and 97.5th percentiles, respectively, and 90% confidence intervals for both limits were calculated using bootstrap resampling [16,17]. Potential outliers were assessed within each partition by visual inspection of the data distribution, and suspected extreme values were reviewed against the original records and eligibility criteria. No additional exclusions were made, as no data entry errors, measurement errors, or violations of the eligibility criteria were identified. Mean ± standard deviation, median with interquartile range, and minimum-maximum values were also calculated for each partition. Univariate linear regression analyses were performed to examine factors associated with SMIP. The candidate variables were age, sex, height, weight, BMI, SMI, %VC, FEV_1_/FVC, and MIP. Sex was coded as 1 for males and 0 for females. Multiple linear regression analysis was then performed to develop a prediction equation for SMIP. The final model included age, sex, height, and weight to create a simple and practical equation based on readily available demographic and anthropometric variables. Model performance was evaluated using the coefficient of determination (R^2^), adjusted R^2^, and the standard error of the estimate (SEE). Multicollinearity was assessed using the variance inflation factor (VIF). A p-value of < 0.05 was considered statistically significant.

## Results

Participant characteristics

**Figure 1:**
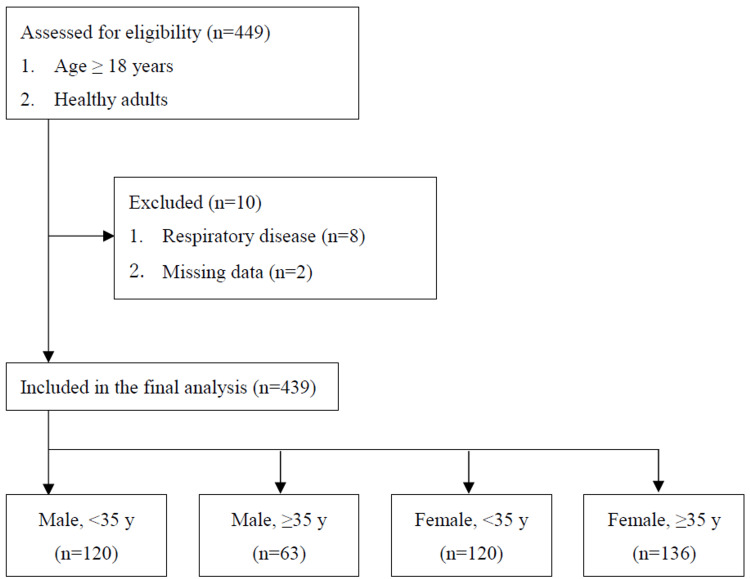
Participant flow diagram

A total of 449 healthy adults aged 18 years or older met the inclusion criteria. The exclusion criteria were the presence of respiratory or neuromuscular disease and missing data. No participants were excluded because of neuromuscular disease. After excluding 10 participants, 439 participants were included in the final analysis. The number of participants in each partition was 120 males aged <35 years, 63 males aged ≥35 years, 120 females aged <35 years, and 136 females aged ≥35 years (Figure 1). The mean age was 21.5 ± 3.6 years in younger males, 59.0 ± 16.4 years in older males, 22.0 ± 4.3 years in younger females, and 67.8 ± 14.0 years in older females. Mean height and MIP were numerically higher in males than in females, and MIP was also numerically higher in younger participants than in older participants (Table 1).

**Table 1:** Participant characteristics according to sex and age group Data are presented as mean ± standard deviation. BMI: body mass index, SMI: skeletal muscle mass index, %VC: percent vital capacity, FEV_1_/FVC: forced expiratory volume in one second as a percentage of forced vital capacity, MIP: maximum inspiratory pressure, SMIP: sustained maximum inspiratory pressure, PTU: pressure time unit.

Variable	Male <35 y	Male ≥35 y	Female <35 y	Female ≥35 y
	(n=120)	(n=63)	(n=120)	(n=136)
Age (years)	21.5±3.6	59.0±16.4	22.0±4.3	67.8±14.0
Height (cm)	172.1±6.1	168.0±8.5	157.9±5.0	153.2±6.6
Weight (kg)	65.8±9.5	66.8±10.1	51.3±6.5	53.6±8.4
BMI (kg/m^2^)	22.2±2.7	23.6±3.1	20.6±2.3	22.8±3.2
SMI (kg/m^2^)	8.0±0.8	7.9±0.8	6.2±0.6	6.3±0.9
%VC (%)	86.3±10.4	90.4±14.1	85.6±9.3	93.7±15.2
FEV_1_/FVC (%)	88.5±6.2	83.3±7.4	89.4±6.6	80.9±9.7
MIP (cmH_2_O)	102.4±26.9	86.5±32.2	67.5±23.4	56.1±22.1

SMIP reference intervals

Mean SMIP values were 746.4 ± 217.7 PTU in males aged <35 years, 557.3 ± 278.1 PTU in males aged ≥35 years, 386.2 ± 135.3 PTU in females aged <35 years, and 290.8 ± 124.1 PTU in females aged ≥35 years. Median (IQR) values were 740 (585-869), 528 (321-717), 385 (286-481), and 280 (211-351), respectively.

Using the percentile method, the 95% reference intervals of SMIP were 383.2-1209.9 PTU for males aged <35 years, 141.0-1222.0 PTU for males aged ≥35 years, 109.5-667.8 PTU for females aged <35 years, and 97.3-617.1 PTU for females aged ≥35 years. The corresponding minimum-maximum values were 162-1454 PTU, 102-1258 PTU, 87-697 PTU, and 46-636 PTU, respectively (Table 2).

**Table 2:** Reference intervals of SMIP according to sex and age group All SMIP values are expressed in pressure-time units (PTU). SMIP: sustained maximum inspiratory pressure, PTU: pressure time unit, SD: standard deviation, IQR: interquartile range, CI: confidence interval.

Variable	Male <35 y	Male ≥35 y	Female <35 y	Female ≥35 y
n	120	63	120	136
Mean±SD	746.4±217.7	557.3±278.1	386.2±135.3	290.8±124.1
Median (IQR)	740 (585-869)	528 (321-717)	385 (286-481)	280 (211-351)
Minimum-Maximum	162-1454	102-1258	87-697	46-636
2.5th percentile	383.2	141.0	109.5	97.3
90% CI for lower limit	162.0-426.8	102.0-194.4	87.0-142.7	53.5-108.4
97.5th percentile	1209.9	1222.0	667.8	617.1
90% CI for upper limit	1117.9-1454.0	1054.5-1258.0	630.6-691.9	546.0-635.3
95% reference interval	383.2-1209.9	141.0-1222.0	109.5-667.8	97.3-617.1

Factors associated with SMIP

In univariate linear regression analyses, age was negatively associated with SMIP (B = -5.230, standardized beta = -0.485, 95% CI: -6.116 to -4.343, p < 0.001). Sex, height, weight, SMI, %VC, FEV1/FVC, and MIP were positively associated with SMIP. Among these variables, MIP showed the strongest standardized association with SMIP (standardized beta = 0.805, 95% CI: 6.177 to 7.097, p < 0.001) (Table 3).

**Table 3:** Factors associated with SMIP SMIP: sustained maximum inspiratory pressure, CI: confidence interval, BMI: body mass index, SMI: skeletal muscle mass index, %VC: percent vital capacity, FEV_1_/FVC: forced expiratory volume in one second as a percentage of forced vital capacity, MIP: maximum inspiratory pressure.

Variable	Unstandardized β	95% CI	Standardized β	p value
Age (years)	-5.23	-6.116 to -4.343	-0.485	<0.001
Sex (male=1, female=0)	345.764	308.514 to 383.014	0.658	<0.001
Height (cm)	17.597	15.841 to 19.354	0.686	<0.001
Weight (kg)	1.084	0.409 to 1.759	0.149	0.002
BMI (kg/m^2^)	0.058	-1.760 to 1.876	0.003	0.95
SMI (kg/m^2^)	139.528	122.843 to 156.214	0.618	<0.001
%VC (%)	2.568	0.677 to 4.458	0.127	0.008
FEV_1_/FVC (%)	5.175	2.365 to 7.985	0.171	<0.001
MIP (cmH_2_O)	6.637	6.177 to 7.097	0.805	<0.001

Prediction equation for SMIP

In the multiple linear regression analysis, age, sex, and height were independently associated with SMIP, whereas weight was not statistically significant after adjustment. Specifically, SMIP decreased with age (B = -2.85, standardized beta = -0.26, 95% CI: -3.63 to -2.06, p < 0.001), was higher in males than in females (B = 209.86, standardized beta = 0.40, 95% CI: 159.91 to 259.80, p < 0.001), and increased with height (B = 6.51, standardized beta = 0.25, 95% CI: 3.77 to 9.26, p < 0.001), whereas weight was not independently associated with SMIP (B = 0.22, standardized beta = 0.03, 95% CI: -0.24 to 0.68, p = 0.346) (Table 4).

**Table 4:** Multiple linear regression model for predicting SMIP SMIP: sustained maximum inspiratory pressure, SE: standard error, CI: confidence interval, VIF: variance inflation factor. Standardized β and VIF are not applicable to the constant term. Model performance: R^2^ = 0.569; adjusted R^2^ = 0.565; SEE = 171.197 PTU. Prediction equation: Predicted SMIP = -556.90 – 2.85 × Age + 209.86 × Sex + 6.51 × Height + 0.22 × Weight.

Variable	Unstandardized β	SE	Standardized β	95% CI	p value	VIF
Constant	-556.9	224.52	-	-998.18 to -115.62	0.014	-
Age (years)	-2.85	0.4	-0.26	-3.63 to -2.06	<0.001	1.37
Sex (male=1, female=0)	209.86	25.41	0.4	159.91 to 259.80	<0.001	2.35
Height (cm)	6.51	1.4	0.25	3.77 to 9.26	<0.001	2.98
Weight (kg)	0.22	0.24	0.03	-0.24 to 0.68	0.346	1.06

The final model explained 56.9% of the variance in SMIP (R^2^ = 0.569; adjusted R^2^ = 0.565), with a SEE of 171.20 PTU. No problematic multicollinearity was observed, with VIF values ranging from 1.06 to 2.98. The resulting prediction equation was as follows:

Predicted SMIP = -556.90 - 2.85 × Age + 209.86 × Sex + 6.51 × Height + 0.22 × Weight

## Discussion

The present study established sex- and age-specific reference intervals for SMIP, identified factors associated with SMIP, and developed a practical prediction equation in healthy adults. SMIP was highest in younger males and lowest in older females, and age, sex, and height emerged as independent predictors in the final model. These findings support the interpretation of SMIP using sex- and age-specific reference intervals rather than a single pooled standard.

A clear gradient in SMIP was observed across the four partitions, with higher values in males and younger participants. This pattern is consistent with previous studies showing that respiratory muscle performance is influenced by sex-related differences in body size and muscle characteristics, as well as aging-related decline [5,7,11,18]. The relatively wide reference interval observed in males aged ≥35 years may reflect greater inter-individual variability, the relatively small sample size in this partition, or a combination of both. Therefore, the reference interval for this subgroup should be interpreted with appropriate caution.

In the univariate analyses, demographic, anthropometric, body composition, pulmonary function variables, and MIP were associated with SMIP. Among these, MIP showed the strongest standardized association. This is physiologically plausible because both MIP and SMIP reflect inspiratory muscle performance [1,2,9]. However, SMIP may offer an important advantage over MIP because it reflects not only peak inspiratory pressure but also the ability to sustain inspiratory effort throughout the maneuver [8,9]. In this sense, SMIP may capture a broader dimension of inspiratory muscle performance than MIP and may therefore provide information that is more relevant to functional capacity than instantaneous inspiratory strength alone [8-10]. This interpretation is also consistent with previous studies reporting associations of skeletal muscle mass with respiratory muscle strength and pulmonary function [19,20].

The final regression model was designed to develop a simple and practical prediction equation rather than the most explanatory model possible. For this reason, age, sex, height, and weight were selected a priori for multivariable analysis, even though body composition and pulmonary function variables were also associated with SMIP in the univariate analyses. Although weight was not independently associated with SMIP after adjustment, it was retained in the final model to preserve the practicality and consistency of the equation. This approach improves usability in routine clinical and research settings, where demographic and anthropometric information is readily available but more detailed assessments may not be feasible. Accordingly, factors associated with SMIP in univariate analyses should be distinguished from the predictors retained in the final practical model.

In the final model, age, sex, and height remained significant independent predictors, whereas weight was not independently associated with SMIP after adjustment. This suggests that the apparent contribution of weight may have been partly explained by shared variance with sex and body size. Age showed a negative association with SMIP, which may reflect age-related declines in skeletal muscle mass, muscle quality, physical function, and neuromuscular performance [4,21,22]. Because SMIP incorporates sustained inspiratory effort, it may be particularly sensitive to such age-related changes. These findings are broadly compatible with previous inspiratory muscle strength studies [5-7,11,17].

The final model explained 56.9% of the variance in SMIP, indicating moderate predictive performance. No problematic multicollinearity was observed, supporting the stability of the model. From a practical perspective, the resulting equation may be useful for estimating expected SMIP values in healthy adults using only easily obtainable variables. At the same time, a substantial proportion of variability remained unexplained, suggesting that other factors, such as habitual physical activity, respiratory muscle coordination, motivation during the maneuver, or unmeasured aspects of body composition, may also influence SMIP.

The present findings have several clinical implications. First, the sex- and age-specific reference intervals may facilitate identification of SMIP values that are lower than expected for a given individual. Second, the prediction equation provides an individualized framework for interpreting measured values. For example, comparison of an individual’s measured SMIP with the predicted SMIP derived from the present equation may help determine whether inspiratory muscle performance is lower than expected for that person’s demographic and anthropometric characteristics. Such an approach may facilitate individualized interpretation of SMIP values in both clinical and research settings. Third, the broader physiological meaning of SMIP, compared with MIP alone, suggests that SMIP may be a useful index when a more comprehensive assessment of inspiratory muscle performance is desired [8-10].

Several limitations should be acknowledged. First, the reference intervals and prediction equation were derived from a single dataset, and no external validation was performed. Second, although nonparametric reference interval estimation generally requires at least 120 individuals in each partition, the male ≥35 years partition included fewer participants, which may have reduced the stability of the estimated lower and upper reference limits [16]. Third, the age cut-off of 35 years was adopted as a practical framework for age-stratified estimation rather than as a biologically definitive threshold for SMIP. Fourth, smoking history, habitual physical activity, obesity, cardiovascular disease, medication use, and other comorbidities were not systematically assessed and may have influenced SMIP. Further stratification according to these additional factors was not feasible because it would have substantially increased the number of partitions and reduced the sample size within each partition, thereby limiting the reliability of reference interval estimation. Fifth, because the final model was intentionally simplified for practical use, it may not fully reflect all determinants of SMIP. Future studies should validate the present equation in independent samples, evaluate the influence of additional health- and lifestyle-related factors, and compare its performance with more comprehensive models.

## Conclusions

In conclusion, this study established sex- and age-specific reference intervals for SMIP and developed a simple and practical prediction equation for healthy adults. Age, sex, and height were identified as independent predictors of SMIP. These findings provide a basis for the quantitative interpretation of SMIP and support its use as a more comprehensive index of inspiratory muscle performance than MIP alone. The proposed reference intervals and prediction equation may facilitate the interpretation of individual SMIP values in both clinical and research settings. In addition, these findings may contribute to the broader use of SMIP in the assessment of inspiratory muscle performance in healthy adults.
